# Supplementary material for: Neoadjuvant–adjuvant pertuzumab in HER2-positive early breast cancer: final analysis of the randomized phase III PEONY trial
Source: Nat Commun. 2024 Mar 9;15:2153. doi: 10.1038/s41467-024-45591-7 (PMC10925021; doi:10.1038/s41467-024-45591-7)
Supplement: Supplementary file 1 — Supplementary Information [file 41467_2024_45591_MOESM1_ESM.pdf]

Supplementary Information

Supplementary Note

The study protocol is available with the previously published primary analysis (DOI: 10.1001/jamaoncol.2019.3692).

Supplementary Methods

List of additional antibodies used in this study

Antibodies used in assays included pertuzumab rhuMAb 2C4 (supplied by Genentech Inc., lot no. 67966-40/anti2C4907-2, diluted to 100 µg/mL stock solution), biotin-rhuMAb 2C4 (supplied by Genentech Inc., lot no. mehrabak-16Jun17-29, diluted to 600 µg/mL stock solution); DIG-rhuMAb 2C4 (supplied by Genentech Inc., lot no. mehrabak-16Jun17-95, diluted to 600 µg/mL stock solution); peroxidase-conjugated IgG fraction monoclonal mouse anti-digoxin (supplied by Jackson Immuno Research, lot no. 126520, 133869, 140839, diluted to 800 µg/mL stock solution); anti-hu2C4 mAb 4290, Clone 19A2.7 (supplied by Genentech Inc., lot no. ikim-14Aug13-48 [PUR47100], dilutions were variable depending on the assays used); PTEN (138G6) rabbit mAb (supplied by Cell Signaling Technology; catalog no. #9559; dilutions were variable depending on the assays used).

Supplementary Table 1 | Baseline demographics and disease characteristics

Demographic/characteristic	Pertuzumab arm (*n* = 219)	Placebo arm (*n* = 110)
Median age, years (range)	49 (24–72)	49 (27–70)
Age, *n* (%)		
<40 years	40 (18.3)	18 (16.4)
40–49 years	75 (34.2)	40 (36.4)
50–64 years	96 (43.8)	44 (40.0)
≥65 years	8 (3.7)	8 (7.3)
Region, *n* (%)		
Mainland China	175 (79.9)	86 (78.2)
Taiwan	18 (8.2)	13 (11.8)
Other	26 (11.9)	11 (10.0)
ECOG Performance Status, *n* (%)		
0	198 (90.4)	97 (88.2)
1	21 (9.6)	13 (11.8)
Hormone receptor status (IxRS), *n* (%)		
ER- and PgR-negative	105 (47.9)	54 (49.1)
ER- and/or PgR-positive	114 (52.1)	56 (50.9)
Menopausal status, *n* (%)		
Premenopausal	132 (60.3)	65 (59.1)
Postmenopausal	87 (39.7)	45 (40.9)
Disease status (IxRS), *n* (%)		
Early	152 (69.4)	77 (70.0)
Locally advanced	67 (30.6)	33 (30.0)
Primary tumor stage, *n* (%)		
T2	155 (70.8)	71 (64.5)
T3	45 (20.5)	29 (26.4)
T4	19 (8.7)	10 (9.1)
Lymph node status, *n* (%)		
Positive	160 (73.1)	89 (80.9)
Negative	59 (26.9)	21 (19.1)
Histologic subtype,[a] *n* (%)		
Ductal	203 (92.7)	103 (93.6)
Lobular	4 (1.8)	1 (0.9)
Comedo	0	1 (0.9)
Other/comedo	15 (6.8)	8 (7.3)
HER2 IHC score, *n* (%)		
1+	2 (0.9)	0
2+	65 (29.7)	22 (20.0)
3+	152 (69.4)	88 (80.0)
Breast surgery, *n* (%)		
Breast-conserving surgery	57 (26.0)	35 (31.8)
Mastectomy	148 (67.6)	70 (63.6)
Other	5 (2.3)	0
Axillary lymph node surgery, *n* (%)		
Sentinel lymph node biopsy	34 (15.5)	17 (15.5)
Axillary lymph node dissection	171 (78.1)	88 (80.0)
Other	5 (2.3)	0
Adjuvant FEC treatment, *n* (%)	208 (95.0)	103 (93.6)
Adjuvant anti-HER2 treatment, *n* (%)	204 (93.2)	99 (90.0)
Radiotherapy, *n* (%)	154 (70.3)	79 (71.8)

[a]More than one histologic subtype could be selected.
ECOG, Eastern Cooperative Oncology Group; ER, estrogen receptor; FEC, 5-fluorouracil, epirubicin, and cyclophosphamide; HER2, human epidermal growth factor receptor 2; IHC, immunohistochemistry; IxRS, interactive voice/web response system; PgR, progesterone receptor.

Supplementary Table 2 | Exposure to pertuzumab or placebo in the adjuvant treatment period (safety-evaluable population)

	Pertuzumab group (*n* = 208)	Placebo group (*n* = 103)
Treatment duration (weeks)		
n	204	99
Mean (SD)	38.9 (2.4)	37.8 (6.2)
Median	39.0	39.0
Min.–max.	15–43	3–42
Number of cycles		
n	204	99
Mean (SD)	12.9 (0.8)	12.6 (2.0)
Median	13.0	13.0
Min.–max.	5–13	1–13
Number of cycles (group)		
n	204	99
1–12	8 (3.8)	5 (4.9)
13	196 (94.2)	94 (91.3)
>13	0	0
Cumulative dose (mg)		
n	204	99
Mean (SD)	5,828.5 (320.1)	5,693.3 (857.4)
Median	5,880.0	5,880.0
Min.–max.	2,520–6,300	840–6,300
Number of infusion modifications, *n* (%)		
n	204	99
0	202 (97.1)	99 (96.1)
1	2 (1.0)	0
2	0	0
>2	0	0
Number of infusion modifications due to adverse event, *n* (%)		
n	204	99
0	202 (97.1)	99 (96.1)
1	2 (1.0)	0
2	0	0
>2	0	0

SD, standard deviation.

Supplementary Table 3 | Exposure to trastuzumab in the adjuvant treatment period (safety-evaluable population)

	Pertuzumab group (*n* = 208)	Placebo group (*n* = 103)
Treatment duration (weeks)		
n	204	99
Mean (SD)	38.9 (2.4)	37.8 (6.2)
Median	39.0	39.0
Min.–max.	15–43	3–42
Number of cycles		
n	204	99
Mean (SD)	12.9 (0.8)	12.6 (2.0)
Median	13.0	13.0
Min.–max.	5–13	1–13
Number of cycles (group)		
n	204	99
1–12	6 (2.9)	5 (4.9)
13	198 (95.2)	94 (91.3)
>13	0	0
Cumulative dose (mg)		
n	204	99
Mean (SD)	4,847.8 (929.2)	4,601.8 (1049.4)
Median	4720.0	4,720.0
Min.–max.	2,016–7,920	382–6,720
Number of infusion modifications, *n* (%)		
n	204	99
0	196 (94.2)	98 (95.1)
1	8 (3.8)	1 (1.0)
2	0	0
>2	0	0
Number of infusion modifications due to adverse event, *n* (%)		
n	204	99
0	199 (95.7)	98 (95.1)
1	5 (2.4)	1 (1.0)
2	0	0
>2	0	0

SD, standard deviation.

Supplementary Table 4 | Exposure to FEC in the adjuvant treatment period (safety-evaluable population)

	5-fluorouracil		Epirubicin		Cyclophosphamide	
	**Pertuzumab group (*n* = 208)**	**Placebo group (*n* = 103)**	**Pertuzumab group (*n* = 208)**	**Placebo group (*n* = 103)**	**Pertuzumab group (*n* = 208)**	**Placebo group (*n* = 103)**
Treatment duration (weeks)						
n	198	101	208	103	208	103
Mean (SD)	8.8 (1.4)	8.9 (1.3)	9.1 (0.8)	9.1 (0.8)	9.1 (0.8)	9.1 (0.8)
Median	9.0	9.0	9.0	9.0	9.0	9.0
Min.–max.	3–13	3–11	3–13	3–11	3–13	3–11
Number of cycles						
n	198	101	208	103	208	103
Mean (SD)	2.9 (0.4)	2.9 (0.4)	3.0 (0.2)	3.0 (0.2)	3.0 (0.2)	3.0 (0.2)
Median	3.0	3.0	3.0	3.0	3.0	3.0
Min.–max.	1–3	1–3	1–3	1–3	1–3	1–3
Number of cycles (group)						
n	198	101	208	103	208	103
1	7 (3.4)	4 (3.9)	1 (0.5)	1 (1.0)	1 (0.5)	1 (1.0)
2	9 (4.3)	3 (2.9)	2 (1.0)	1 (1.0)	2 (1.0)	1 (1.0)
3	182 (87.5)	94 (91.3)	205 (98.6)	101 (98.1)	205 (98.6)	101 (98.1)
>3	0	0	0	0	0	0
Cumulative dose (mg)						
n	198	101	208	103	208	103
Mean (SD)	2,388.6 (454.9)	2,384.7 (433.2)	447.5 (53.5)	438.8 (52.2)	2,522.1 (343.6)	2,482.9 (328.1)
Median	2,400.0	2,400.0	450.0	442.0	2,475.0	2,475.0
Min.–max.	612–3,564	850–3,150	110–603	170–564	612–3,600	850–3,150
Number of dose modifications, *n* (%)						
n	198	101	208	103	208	103
0	176 (84.6)	96 (93.2)	182 (87.5)	92 (89.3)	171 (82.2)	92 (89.3)
1	21 (10.1)	2 (1.9)	23 (11.1)	8 (7.8)	35 (16.8)	5 (4.9)
2	1 (0.5)	2 (1.9)	3 (1.4)	3 (2.9)	2 (1.0)	5 (4.9)
>2	0	1 (1.0)	0	0	0	1 (1.0)
Number of dose modifications due to AE, *n* (%)						
n	198	101	208	103	208	103
0	182 (87.5)	98 (95.1)	189 (90.9)	95 (92.2)	191 (91.8)	98 (95.1)
1	15 (7.2)	3 (2.9)	17 (8.2)	7 (6.8)	16 (7.7)	5 (4.9)
2	1 (0.5)	0	2 (1.0)	1 (1.0)	1 (0.5)	0
>2	0	0	0	0	0	0

AE, adverse event; FEC, 5-fluorouracil, epirubicin, and cyclophosphamide; SD, standard deviation.

Supplementary Table 5 | Death by cause in the safety-evaluable population

**Patients, *n* (%)**	**Pertuzumab group (*n* = 218)**	**Placebo group (*n* = 110)**
All deaths	12 (5.5)	11 (10.0)
Other	2 (0.9)	2 (1.8)
Progression of disease	7 (3.2)	5 (4.5)
Recurrence of disease	1 (0.5)	2 (1.8)
Adverse event	2 (0.9)	2 (1.8)
Neoadjuvant study treatment period	1 (0.5)	0
Other	0	0
Progression of disease	0	0
Recurrence of disease	0	0
Adverse event	1 (0.5)	0
Treatment-free follow-up	10 (4.6)	10 (9.1)
Other	2 (0.9)	1 (0.9)
Progression of disease	6 (2.8)	5 (4.5)
Recurrence of disease	1 (0.5)	2 (1.8)
Adverse event	1 (0.5)	2 (1.8)
Death date is missing/wrong	1 (0.5)	1 (0.9)
Other	0	1 (0.9)
Progression of disease	1 (0.5)	0
Recurrence of disease	0	0
Adverse event	0	0

Supplementary Table 6 | List of ethics committees (EC)

Ethics committees of institutes participating in this study	EC number
EC of Fudan University Shanghai Cancer Center	N/A
EC of The Cancer Hospital of Harbin Medical University	N/A
IEC of Zhejiang Cancer Hospital	N/A
The Ethics Committee of The First Hospital of Jilin University	N/A
EC of Sun Yat-sen University Cancer Center	N/A
Ethics committee of Henan Tumor Hospital	N/A
EC of Guangdong General Hospital	N/A
IEC of Shandong Cancer Hospital	N/A
Fujian Medical University Union Hospital; Ethics Committee	N/A
MacKay Memorial Hospital Institutional Review Board	14CT013b
Research Ethics Committee China Medical University & Hospital	CMUH103-REC2-109
Ajou University Hospital; IRB	N/A
Korea University Guro Hospital; Oncology; IRB	N/A
EC of Ruijin Hospital, Shanghai Jiao Tong University School of Medicine	N/A
Central Research EC of Prince of Songkla University	N/A
307 Hospital Ethics Committee	N/A

Supplementary Fig. 1 | Kaplan–Meier plot of OS in the ITT population.

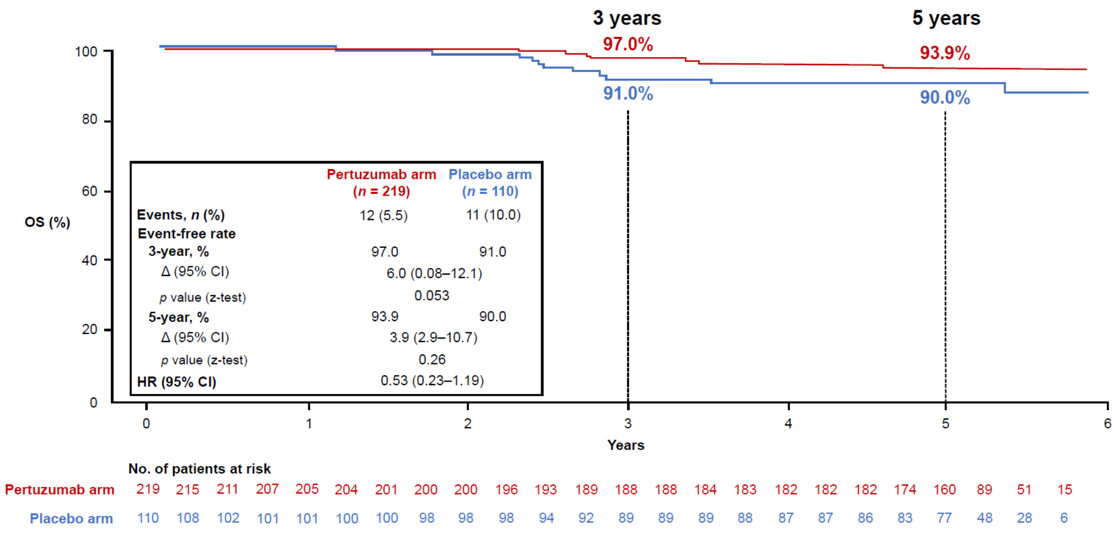

95% CIs and *p* values (two-sided) for differences in event rates were from z-tests using the standard errors for the Kaplan–Meier estimates. CI, confidence interval; HR, hazard ratio; ITT, intention to treat; OS, overall survival.

Supplementary Fig. 2 | Subgroup analysis of EFS according to treatment arm in the ITT population.

Baseline risk factors	Total, *n*	Placebo + trastuzumab + chemotherapy (*n* = 110) *n*	Events	Median, months	Pertuzumab + trastuzumab + chemotherapy (*n* = 219) *n*	Events	Median, months	Hazard ratio	95% Wald CI
**All patients**	329	110	27	NE	219	32	NE	0.53	0.32–0.89
**Disease stage**									0.30–1.25
Early stage	228	76	14	NE	152	17	NE	0.62	
Locally advanced	101	34	13	NE	67	15	NE	0.41	0.19–0.88
**Hormone receptor status**									
ER- and PgR-negative	156	54	17	NE	102	15	NE	0.44	0.22–0.89
ER- and/or PgR-positive	173	56	10	NE	117	17	NE	0.70	0.32–1.53
**Age (years)**									
<65	313	102	26	NE	211	32	NE	0.52	0.31–0.87
≥65	16	8	1	NE	8	0	NE	<0.01	0.00–NE
**Region**									
Mainland of China	261	86	22	NE	175	27	NE	0.57	0.32–1.00
Other	37	11	4	NE	26	4	NE	0.27	0.6–1.25
Taiwan	31	13	1	NE	18	1	NE	0.71	0.04–11.79
**Menopausal status**									
Postmenopausal	131	45	8	NE	86	12	NE	0.70	0.28–1.72
Premenopausal	196	65	19	NE	133	20	NE	0.43	0.23–0.82
**Primary tumor stage**									
T2	228	71	17	NE	157	17	NE	0.41	0.21–0.81
T3 or larger	101	39	10	NE	62	15	NE	0.74	0.33–1.67
**Lymph node status**									
Negative	81	22	2	NE	59	3	NE	0.36	0.06–2.20
Positive	248	88	25	NE	160	29	NE	0.57	0.33–0.97
**Histologic subtype**									
Ductal	306	103	26	NE	203	30	NE	0.51	0.30–0.86
Non-ductal	23	7	1	NE	16	2	NE	0.58	0.03–10.25
**Targeted surgery type**									
Breast conserving	92	35	7	NE	57	7	NE	0.37	0.12–1.16
Mastectomy	218	70	18	NE	148	22	NE	0.54	0.29–1.01
Other	5				5	0	NE	NE	NE-NE
**HER2 IHC result**									
2+	87	22	7	NE	65	13	NE	0.47	0.18–1.23
3+	240	88	20	NE	152	19	NE	0.46	0.24–0.87

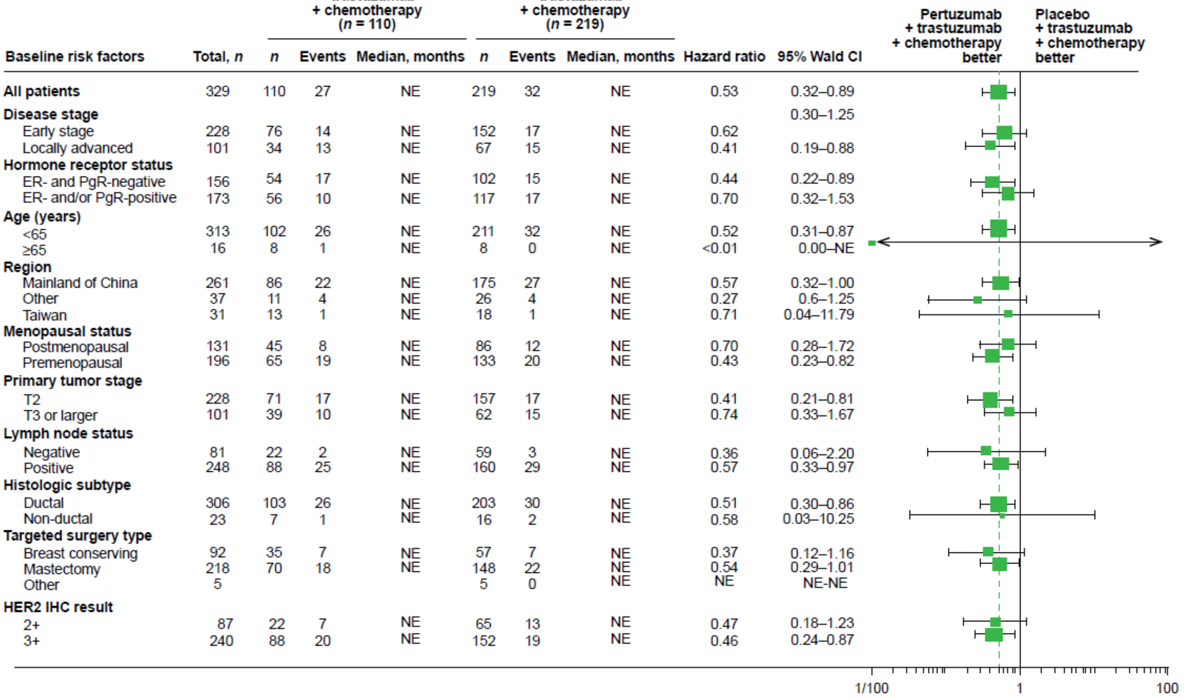

Boxes represent sample sizes and hazard ratios; whiskers represent 95% CI; the vertical dotted line represents the hazard ratio for all patients. CI, confidence interval; EFS, event-free survival; ER, estrogen receptor; FISH, fluorescence *in situ* hybridization; HER2, human epidermal growth factor receptor 2; IHC, immunohistochemistry; ITT, intention-to-treat; NE, not evaluable; PgR, progesterone receptor.

Supplementary Fig. 3 | Subgroup analysis of DFS according to treatment arm in the ITT population.

		Placebo + trastuzumab + chemotherapy (*n* = 105)			Pertuzumab + trastuzumab + chemotherapy (*n* = 210)				
**Baseline risk factors**	**Total, *n***	***n***	**Events**	**Median, months**	***n***	**Events**	**Median, months**	**Hazard ratio**	**95% Wald CI**
**All patients**	315	105	25	NE	210	29	NE	0.52	0.30–0.88
**Disease stage**									
Early stage	223	74	13	NE	149	17	NE	0.67	0.32–1.38
Locally advanced	92	31	12	NE	61	12	NE	0.33	0.15–0.75
**Hormone receptor status**									
ER- and PgR-negative	149	52	16	NE	97	13	NE	0.40	0.19–0.85
ER- and/or PgR-positive	166	53	9	NE	113	16	NE	0.73	0.32–1.65
**Age (years)**									
<65	300	98	24	NE	202	29	NE	0.51	0.29–0.87
≥65	15	7	1	NE	8	0	NE	<0.01	0.00–NE
**Region**									
Mainland of China	249	81	20	NE	168	25	NE	0.59	0.32–1.06
Other	35	11	4	NE	24	3	NE	0.18	0.03–1.02
Taiwan	31	13	1	NE	18	1	NE	0.71	0.04–11.79
**Menopausal status**									
Postmenopausal	127	44	8	NE	83	11	NE	0.65	0.26–1.62
Premenopausal	188	61	17	NE	127	18	NE	0.43	0.22–0.83
**Primary tumor stage**									
T2	223	70	16	NE	153	17	NE	0.44	0.22–0.88
T3 or larger	92	35	9	NE	57	12	NE	0.61	0.25–1.48
**Lymph node status**									
Negative	79	21	2	NE	58	3	NE	0.36	0.06–2.20
Positive	236	84	23	NE	152	26	NE	0.55	0.31–0.98
**Histologic subtype**									
Ductal	294	99	25	NE	195	27	NE	0.47	0.27–0.82
Non-ductal	21	6	0	NE	15	2	NE	>999.99	0.00–NE
**Targeted surgery type**									
Breast conserving	92	35	7	NE	57	7	NE	0.37	0.12–1.16
Mastectomy	218	70	18	NE	148	22	NE	0.54	0.29–1.02
Other	5				5	0	NE	NE	NE-NE
**HER2 IHC result**									
2+	83	20	5	NE	63	12	NE	0.65	0.22–1.91
3+	230	85	20	NE	145	17	NE	0.41	0.21–0.79
**tpCR Status**									
No/missing	205	81	24	NE	124	22	NE	0.56	(0.31–1.00)
Yes	110	24	1	NE	86	7	NE	2.02	(0.25–16.47)

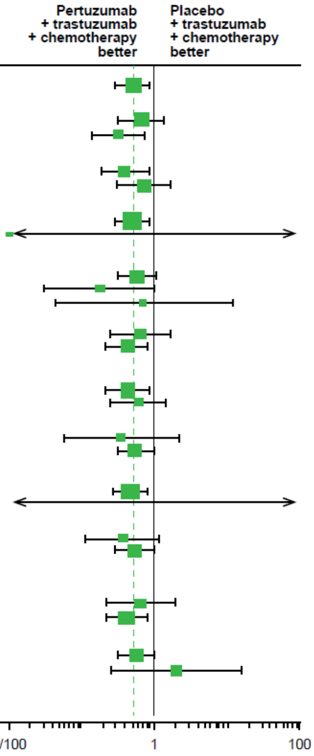

Boxes represent sample sizes and hazard ratios; whiskers represent 95% CI; the vertical dotted line represents the hazard ratio for all patients. CI, confidence interval; DFS, disease-free survival; ER, estrogen receptor; FISH, fluorescence *in situ* hybridization; HER2, human epidermal growth factor receptor 2; IHC, immunohistochemistry; ITT, intention-to-treat; NE, not evaluable; PgR, progesterone receptor; tpCR, total pathologic complete response.

Supplementary Fig. 4 | Biomarker subgroup analysis of EFS rates by HER2 IHC and *PIK3CA* baseline biomarker status in the ITT population.

		HER2 IHC 1+/2+ (*n* = 89)			HER2 IHC 3+ (*n* = 240)				
Baseline risk factors	Total, *n*	*n*	Events	Median, days	*n*	Events	Median, days	Hazard ratio	95% Wald CI
All patients	329	89	20	NE	240	39	NE	0.70	0.41–1.20

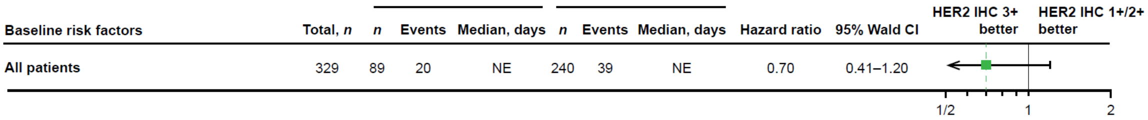

		PIK3CA-mutated (*n* = 98)			*PIK3CA* non-mutated (*n* = 230)				
Baseline risk factors	Total, *n*	*n*	Events	Median, days	*n*	Events	Median, days	Hazard ratio	95% Wald CI
All patients	328	98	27	NE	230	32	NE	0.45	0.27–0.75

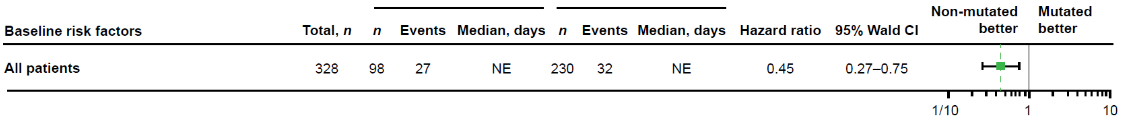

Boxes represent sample sizes and hazard ratios; whiskers represent 95% CI; the vertical dotted line represents the hazard ratio for all patients. CI, confidence interval; EFS, event-free survival; HER2, human epidermal growth factor receptor 2; IHC, immunohistochemistry; ITT, intention to treat; NE, not evaluable; *PIK3CA*, phosphatidylinositol-4,5-bisphosphate 3-kinase catalytic subunit alpha.

Supplementary Fig. 5 | Biomarker subgroup analysis of DFS rates by HER2 IHC and *PIK3CA* baseline biomarker status in the ITT population.

		HER2 IHC 1+/2+ (*n* = 89)			HER2 IHC 3+ (*n* = 240)				
Baseline risk factors	Total, *n*	*n*	Events	Median, days	*n*	Events	Median, days	Hazard ratio	95% Wald CI
All patients	315	85	17	NE	230	37	NE	0.78	0.44–1.38

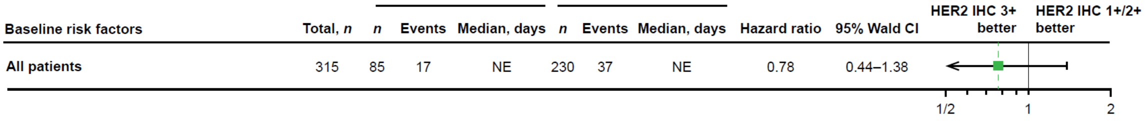

		PIK3CA-mutated (*n* = 98)			*PIK3CA* non-mutated (*n* = 230)				
Baseline risk factors	Total, *n*	*n*	Events	Median, days	*n*	Events	Median, days	Hazard ratio	95% Wald CI
All patients	314	91	24	NE	223	30	NE	0.48	0.28–0.81

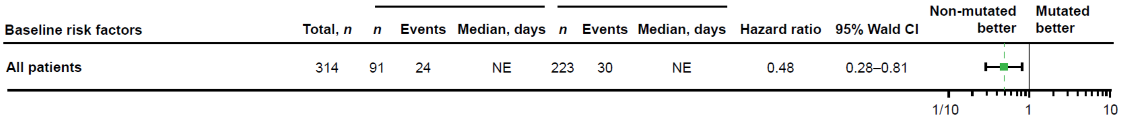

Boxes represent sample sizes and hazard ratios; whiskers represent 95% CI; the vertical dotted line represents the hazard ratio for all patients. CI, confidence interval; DFS, disease-free survival; HER2, human epidermal growth factor receptor 2; IHC, immunohistochemistry; ITT, intention to treat; NE, not evaluable; *PIK3CA*, phosphatidylinositol-4,5-bisphosphate 3-kinase catalytic subunit alpha.

Supplementary Fig. 6 | PEONY study design.

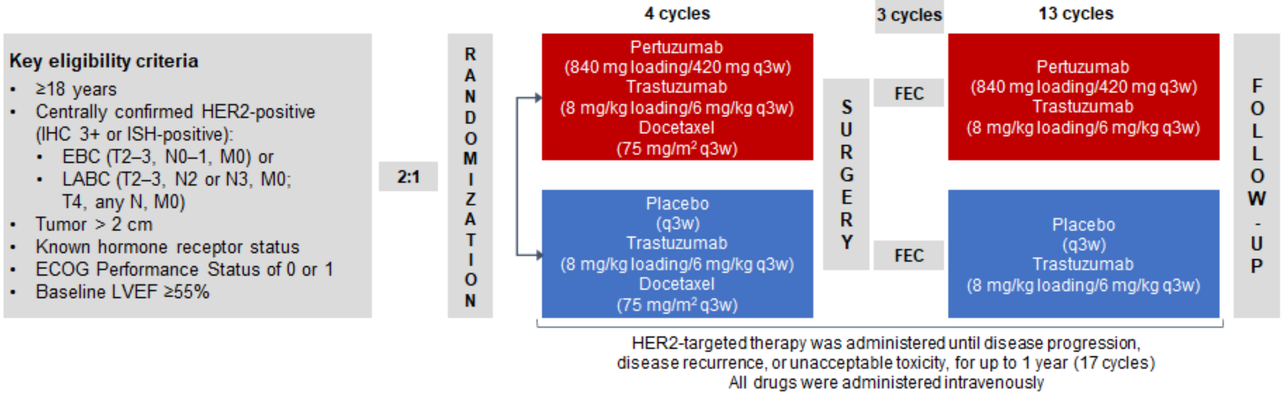

EBC, early breast cancer; ECOG, Eastern Cooperative Oncology Group; FEC, 5-fluorouracil, epirubicin, and cyclophosphamide; HER2, human epidermal growth factor receptor 2; IHC, immunohistochemistry; ISH, *in situ* hybridization; LABC, locally advanced breast cancer; LVEF, left ventricular ejection fraction; q3w, every 3 weeks.
