# Supplementary material for: Neoadjuvant–adjuvant pertuzumab in HER2-positive early breast cancer: final analysis of the randomized phase III PEONY trial
Source: Nat Commun. 2024 Mar 9;15:2153. doi: 10.1038/s41467-024-45591-7 (PMC10925021; doi:10.1038/s41467-024-45591-7)
Supplement: Supplementary file 3 — Reporting Summary [file 41467_2024_45591_MOESM3_ESM.pdf]

nature portfolio

Corresponding author(s): Zhimin Shao

Last updated by author(s): Dec 7, 2023

Reporting Summary

Nature Portfolio wishes to improve the reproducibility of the work that we publish. This form provides structure for consistency and transparency in reporting. For further information on Nature Portfolio policies, see our Editorial Policies and the Editorial Policy Checklist.

Statistics

For all statistical analyses, confirm that the following items are present in the figure legend, table legend, main text, or Methods section.

n/a	Confirmed	
☐	☒	The exact sample size (*n*) for each experimental group/condition, given as a discrete number and unit of measurement
☒	☐	A statement on whether measurements were taken from distinct samples or whether the same sample was measured repeatedly
☐	☒	The statistical test(s) used AND whether they are one- or two-sided<br>*Only common tests should be described solely by name; describe more complex techniques in the Methods section.*
☐	☒	A description of all covariates tested
☒	☐	A description of any assumptions or corrections, such as tests of normality and adjustment for multiple comparisons
☐	☒	A full description of the statistical parameters including central tendency (e.g. means) or other basic estimates (e.g. regression coefficient) AND variation (e.g. standard deviation) or associated estimates of uncertainty (e.g. confidence intervals)
☐	☒	For null hypothesis testing, the test statistic (e.g. *F*, *t*, *r*) with confidence intervals, effect sizes, degrees of freedom and *P* value noted<br>*Give P values as exact values whenever suitable.*
☒	☐	For Bayesian analysis, information on the choice of priors and Markov chain Monte Carlo settings
☒	☐	For hierarchical and complex designs, identification of the appropriate level for tests and full reporting of outcomes
☒	☐	Estimates of effect sizes (e.g. Cohen's *d*, Pearson's *r*), indicating how they were calculated

Our web collection on statistics for biologists contains articles on many of the points above.

Software and code

Policy information about availability of computer code

Data collection	Data entered manually were collected via a sponsor-designated electronic data capture system with the use of electronic case report forms, and was carried out by each participating study site.
Data analysis	Analyses were conducted using SAS v9.4 (SAS Institute, Inc. [Cary, CA]).

For manuscripts utilizing custom algorithms or software that are central to the research but not yet described in published literature, software must be made available to editors and reviewers. We strongly encourage code deposition in a community repository (e.g. GitHub). See the Nature Portfolio guidelines for submitting code & software for further information.

Data

Policy information about availability of data

All manuscripts must include a data availability statement. This statement should provide the following information, where applicable:

- Accession codes, unique identifiers, or web links for publicly available datasets
- A description of any restrictions on data availability
- For clinical datasets or third party data, please ensure that the statement adheres to our policy

The data supporting the findings of this study cannot be made available openly owing to their proprietary nature. Qualified researchers may request access to individual patient-level data through the clinical study data request platform: https://vivli.org/. Further details of Roche's criteria for eligible studies are available here: https://vivli.org/members/ourmembers/. For further details on Roche's Global Policy on the Sharing of Clinical Information and how to request access to related clinical study documents, see here: https://www.roche.com/research_and_development/who_we_are_how_we_work/clinical_trials/

our_commitment_to_data_sharing.htm.

Research involving human participants, their data, or biological material

Policy information about studies with human participants or human data. See also policy information about sex, gender (identity/presentation), and sexual orientation and race, ethnicity and racism.

Reporting on sex and gender	All patients in the study were female; recruitment was open to male and female participants. No sex-/gender-based analyses were performed as all participants were female.
Reporting on race, ethnicity, or other socially relevant groupings	Participants were randomised with the intention to balance age, and region (mainland China, Taiwan, other) between treatment arms.
Population characteristics	Median age of patients in both treatment arms was 49 years. Randomization was stratified by disease category (early-stage or locally advanced) and hormone receptor status (positive for ER and/or PgR, or negative for both). 69.4%/30.6% of patients in the pertuzumab arm had early/locally advanced disease, compared with 70.0%/30.0% in the placebo arm. 47.9% of patients had ER- and PgR-negative disease and 52.1% had ER- and/or PgR-positive disease in the pertuzumab arm, compared with 49.1% and 50.9%, respectively, in the placebo arm.
Recruitment	Investigators at each site understood the study inclusion/exclusion criteria. If the patient was eligible as considered by the investigator (e.g. meeting the criteria of eBC disease status, ECOG performance, etc.), the candidate patient signed an ICF. After signing the ICF, the patient received a unique patient identification number to ensure confidentiality. Patients were enrolled using a permuted block randomisation procedure using stratification factors. The investigator and sponsor were blinded throughout the process and no self-selection bias or other biases were present.
Ethics oversight	The study was conducted in full accordance with the principles of the Declaration of Helsinki and the International Conference on Harmonisation E6 guideline for Good Clinical Practice. Approval for the protocol and amendments was obtained from an institutional review board and/or independent ethics committees (ethics committees belonged to specific participating sites/hospitals and are listed in the Supplementary Information). All patients provided written informed consent.

Note that full information on the approval of the study protocol must also be provided in the manuscript.

Field-specific reporting

Please select the one below that is the best fit for your research. If you are not sure, read the appropriate sections before making your selection.

[x] Life sciences   [ ] Behavioural & social sciences   [ ] Ecological, evolutionary & environmental sciences

For a reference copy of the document with all sections, see nature.com/documents/nr-reporting-summary-flat.pdf

Life sciences study design

All studies must disclose on these points even when the disclosure is negative.

Sample size	A total of 328 patients have been planned to be randomized into the study in a ratio of 2:1 to pertuzumab (Arm A) or placebo (Arm B), respectively. This will provide 85% power to detect an absolute increase in tpCR rate of 15% in the pertuzumab arm compared with the placebo arm at a two-sided significance level of 5%, assuming the tpCR rate is 20% in the placebo arm.
Data exclusions	Data exclusions are described in the primary manuscript CONSORT diagram (Figure 1). Data were excluded for 54 participants who were excluded as they did not meet the pre-defined, per-protocol study eligibility criteria.<br>The DFS analysis was based on the patients who received surgery, with the rationale being: per protocol, disease-free survival (DFS) was defined as the time from the first date of no disease (i.e. date of surgery) to the first documentation of one of the following events: disease recurrence (local, regional, distant, or contralateral) after surgery; death from any cause.<br>The safety analysis population excludes one patient who did not receive any treatment: per protocol and SAP, the safety population was defined as patients who received any amount of study treatment.<br>Patients who received any amount of pertuzumab were analyzed as part of treatment Arm A, even if pertuzumab was given in error. Patients who were randomized to the study but who do not receive any study drug were not included in the safety population.
Replication	Findings were not replicated or reproduced as these are clinical trial data (disease and/or safety events)
Randomization	After written informed consent has been obtained and eligibility has been established and approved, the study site will obtain the patient randomization number and treatment assignment from the interactive voice/web–based response system (IxRS). Patients should receive their first dose of study treatment on the day of randomization, if possible, but no later than 5 business days after randomization. Patients will be randomized in a 2:1 ratio to one of the two treatment arms (pertuzumab or placebo) through use of the IxRS. Patients will be enrolled using a permuted block randomization procedure that uses the following stratification factors:

	• Disease category: early-stage (T2-3, N0-1, M0) or locally advanced (T2-3, N2 or N3, M0; T4, any N, M0)<br>• Hormone-receptor status: positive for ER and/or PgR; negative for both
Blinding	The investigator and the patient will be blinded to the treatment assignment. All other individuals who are directly involved in this study will remain blinded to the treatment assignment until completion of the primary analysis. Patient treatment assignment will not be unblinded until after the final analysis.

Reporting for specific materials, systems and methods

We require information from authors about some types of materials, experimental systems and methods used in many studies. Here, indicate whether each material, system or method listed is relevant to your study. If you are not sure if a list item applies to your research, read the appropriate section before selecting a response.

Materials & experimental systems

n/a	Involved in the study	
☐	☒	Antibodies
☒	☐	Eukaryotic cell lines
☒	☐	Palaeontology and archaeology
☒	☐	Animals and other organisms
☐	☒	Clinical data
☒	☐	Dual use research of concern
☒	☐	Plants

Methods

n/a	Involved in the study	
☒	☐	ChIP-seq
☒	☐	Flow cytometry
☒	☐	MRI-based neuroimaging

Antibodies

Antibodies used	Trastuzumab and pertuzumab antibodies used in the study are licensed drugs and commercially available: https://www.ema.europa.eu/en/medicines/human/EPAR/herceptin#assessment-history-section and https://www.ema.europa.eu/en/medicines/human/EPAR/perjeta.<br>Antibodies used in assays included pertuzumab rhuMAb 2C4 (supplied by Genentech Inc., lot no. 67966-40/anti2C4907-2, diluted to 100 µg/mL stock solution), biotin-rhuMAb 2C4 (supplied by Genentech Inc., lot no. mehrabak-16Jun17-29, diluted to 600 µg/mL stock solution); DIG-rhuMAb 2C4 (supplied by Genentech Inc., lot no. mehrabak-16Jun17-95, diluted to 600 µg/mL stock solution); peroxidase-conjugated IgG fraction monoclonal mouse anti-digoxin (supplied by Jackson Immuno Research, lot no. 126520, 133869, 140839, diluted to 800 µg/mL stock solution); anti-hu2C4 mAb 4290, Clone 19A2.7 (supplied by Genentech Inc., lot no. ikim-14Aug13-48 [PUR47100], dilutions were variable depending on the assays used); PTEN (138G6) rabbit mAb (supplied by Cell Signaling Technology; catalog no. #9559; dilutions were variable depending on the assays used).
Validation	Trastuzumab and pertuzumab antibodies used in the study are licensed drugs and commercially available: https://www.ema.europa.eu/en/documents/variation-report/herceptin-h-c-278-ii-0047-epar-assessment-report-variation_en.pdf and https://www.ema.europa.eu/en/documents/assessment-report/perjeta-epar-public-assessment-report_en.pdf. HER2 IHC used the Daka Herceptest; positive and negative control tissue were prepared per the manufacturer protocol (https://www.agilent.com/cs/library/packageinsert/public/Copy%20of%20PD04084US_04.pdf); the control slides for staining procedures were provided by Dako. PTEN IHC used PTEN (138G6) Rabbit mAb #9559 from Cell Signaling Technology (manufacturer certificate of analysis https://media.cellsignal.com/coa/9559/19/9559-lot-19-coa.pdf). HER3 (7.3.8) was analysed using the Ventana platform (https://www.ncbi.nlm.nih.gov/pmc/articles/PMC5642894/).

Clinical data

Policy information about clinical studies

All manuscripts should comply with the ICMJEguidelines for publication of clinical research and a completedCONSORT checklist must be included with all submissions.

Clinical trial registration	ClinicalTrials.gov identifier: NCT02586025
Study protocol	https://cdn.jamanetwork.com/ama/content_public/journal/oncology/938409/cbr190010supp1_prod.pdf?Expires=1692976855&Signature=w8WRYypaXeJoNJznaM0CnKxQzhdEMh4XQIb2h~Su-ddSK88iCCCn~A2MS29m~y9djcEBrQPtXU7yIeTIiD6k4~RKk-mLcdjaIZPbnxwL5I~DNlGdKu49m-DgBitcOLnJ4PnUnMftlOn79cmiDlMILwpQ26iQ~JLMLeFE4l2FHLMrrhk6r0VSBM50jKQZQrl7cTyCo2VeZsQtYRFUOlH5YpsXDoOdhf~7Y1g0NA3xNIBvLT8ZS2qDCHGx9FuU47xVQK2PqKEFZlG5B3lI8mz6ysmNJ3s15rXvOxZFX0OCyaqpb9YlWRfm2PHqzDG1qToUNWGY1e44vAacG9P8GjyX8w__&Key-Pair-Id=APKAIE5G5CRDK6RD3PGA
Data collection	Patients were recruited between March 14, 2016 and March 13, 2017. The last patient last visit date and clinical cut-off was March 14, 2022. Data entered manually were collected via electronic data capture using electronic case report forms.<br>Sites were responsible for data entry into the electronic data capture system.
Outcomes	The primary efficacy outcome measure of PEONY (reported in a previous manuscript) was independent review committee-assessed tpCR (i.e. ypT0/is, ypN0 according to the current American Joint Committee on Cancer staging system).<br>In the current manuscript, long-term efficacy endpoints of EFS, DFS, and OS, as well as safety, are reported:<br>EFS was defined as the time from randomization to first documentation of disease progression before surgery (determined according to the Response Evaluation Criteria In Solid Tumors v1.1, excluding contralateral disease in situ), disease recurrence after surgery (local, regional, distant, or contralateral, second primary non-breast cancer), or death by any cause. DFS was defined as the time from surgery to the first documented disease recurrence after surgery (local, regional, distant, or contralateral, second primary non-breast cancer), or death by any cause. OS was defined as the time from randomization to death from any cause. Severity of AEs was graded according to the National Cancer Institute's Common Terminology Criteria for AEs v4.019.
